# Staudinger ligation towards cyclodextrin dimers in aqueous/organic media. Synthesis, conformations and guest-encapsulation ability

**DOI:** 10.3762/bjoc.10.73

**Published:** 2014-04-03

**Authors:** Malamatenia D Manouilidou, Yannis G Lazarou, Irene M Mavridis, Konstantina Yannakopoulou

**Affiliations:** 1 Institute of Advanced Materials, Physicochemical Processes, Nanotechnology & Microsystems, National Center for Scientific Research “Demokritos”, Terma Patriarchou Gregoriou & Neapoleos, Aghia Paraskevi Attikis, 15310 Greece. Tel. +30210 6503796, Fax: +30 210 6511766

**Keywords:** calculations, conformations, cyclodextrin, dimer, inclusion, PM3, Staudinger ligation

## Abstract

β-Cyclodextrin (β-CD) dimers have been prepared using the bioorthogonal Staudinger ligation for the first time. In addition to a known linker, methyl 2-(diphenylphosphanyl)terephthalate, a doubly active linker was specifically developed that enabled connection of two β-CD units in a single step and in aqueous/organic media, under mild conditions and with good yields. A three-carbon spacer between the β-CD torus and the azido group was required for facile dimer formation. The products, as studied by NMR spectroscopy, were found to adopt closed conformations by intramolecular self-inclusion. On the other hand, association via intermolecular binding was also observed in aqueous solution, confirmed by DOSY NMR experiments. Despite self-inclusion, the β-CD cavities were capable of guest encapsulation, as shown by titration experiments: the binding constant with 1-adamantylamine was similar to that of natural β-CD. Theoretical calculations for isolated molecules (PM3 level of theory) and in the presence of solvent [water, PM3(COSMO)] as well as DFT calculations suggested that the compounds prefer to adopt conformations which bring the phenyl groups either inside the β-CD cavity (inclusion) or over its narrow side (vicinal). Thus, Staudinger ligation could be the method of choice for linking CDs exhibiting (i) ease of preparation in aqueous media, in short steps, under mild conditions and in good yields, (ii) satisfactory aqueous solubility and independent binding capacity of the cavities.

## Introduction

**Scheme 1:**
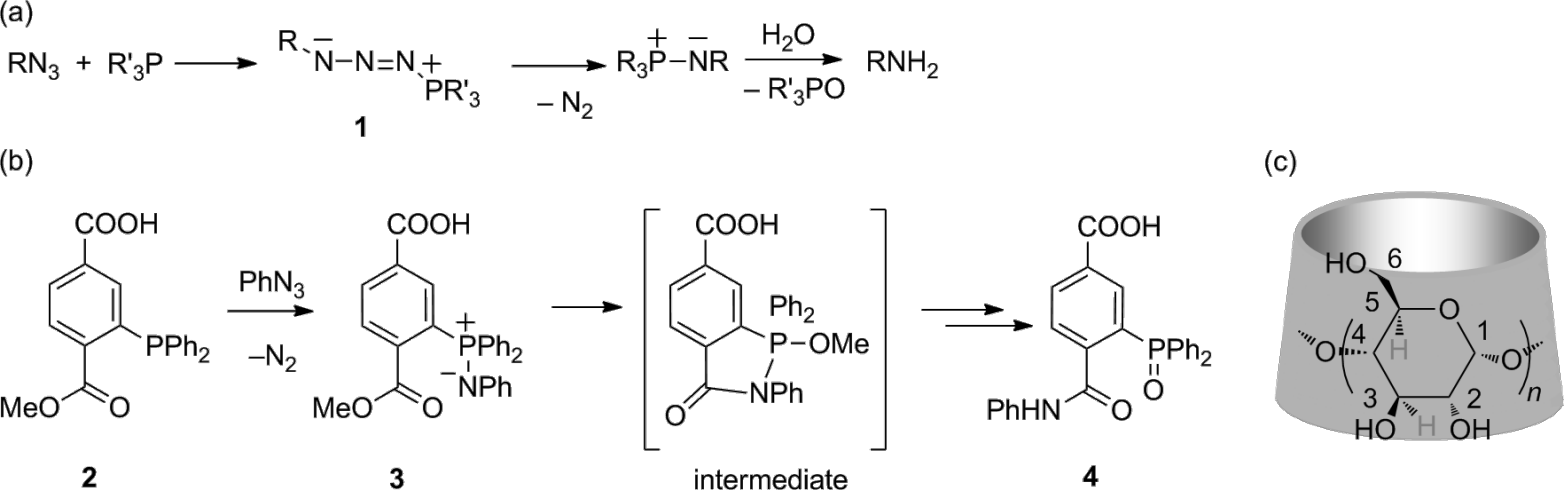
(a) Staudinger reaction (b) Staudinger ligation, (c) the cyclodextrin structure with glucopyranose unit numbering: *n* = 7, β-CD; H3 and H5 atoms are located inside the cavity.

The Staudinger reaction [1] is a classical method for the preparation of amines from phosphines and azides [2–3]. The intermediate, a phosphaza ylide (**1**, Scheme 1a) is readily formed with loss of nitrogen gas, while subsequent hydrolysis yields the corresponding amine and phosphine oxide. A variant of this reaction is the Staudinger ligation [4], a bioorthogonal reaction that has become an important tool of chemical biology in the last decade [5–6]. Introduced by Bertozzi and co-workers, the concept of ligation was based on the design of an intramolecular electrophilic trap, such as the ester carbonyl in methyl terephthalate **2** (Scheme 1b), that upon encounter of an azide (e.g. PhN_3_) can capture the nucleophilic nitrogen of the resulting phosphaza ylide (**3**). After methanol loss and hydrolysis the reaction proceeds to amide bond formation with concomitant phosphine oxidation and formation of **4** in aqueous environment. The ligation proceeds via a cyclic intermediate (Scheme 1b) which has been isolated and its X-ray structure solved in the case of reaction between benzyl azide and 2-(diphenylphosphanyl)benzoic acid methyl ester [7].

The advantages of the Staudinger ligation and its “traceless” variant [6,8] are the employment of the azido group, a moiety orthogonal to naturally existing functional groups and readily introduced into many and different “soft” substrates, the mild reaction conditions, the high yields of the products, and the absence of a catalyst. Numerous applications of linker **2** involving labeling of biomolecules with recognition sites in aqueous media in vitro and on live cells and animals in vivo have been demonstrated [6].

Cyclodextrins (CDs, Scheme 1c), are cyclic oligomers of glucopyranose that act as hosts to hydrophobic molecules in aqueous environment [9–10]. CDs have been recognized as potent drug solubilizers and transporters through biological barriers with increasingly important applications [11]. A specific category of modified CDs are the CD oligomers, compounds with two or three CD macrocycles linked together at different positions (2–2’, 3–3’, 3–2’, 6–2’ etc) via selected spacers. The multicavity structures can, in principle, be precisely tailored to fit specific guest molecules. The oligomers are reported to display improved to significantly enhanced binding capacity as well as superior molecular recognition ability [12–14], compared to the natural CDs. Suitable design has resulted in better hosts for applications as sensors and catalysts [15], hosts of photoactive ligands [16–17], enzyme mimics [18–19], among others, although the synthesis was frequently challenging and elaborate. On another approach, the increased molecular size of oligomers may be advantageous for drug formulations due to foreseen increased circulation time and EPR (Enhanced Permeation and Retention) effect [20], and the ability to carry increased payload, compared to natural CDs. Only few examples of CD oligomers have been studied as hosts to drugs [21]. On a practical point of view, on the other hand, not many reactions have been efficiently applied to produce CD oligomers. These include the well-known copper-catalyzed azide–alkyne cyclization (CuAAC, “click” reaction) between an azido-CD derivative and an alkyne linker [19,22–24] [22], or vice versa, the metal catalyzed reactions between propargyl-CDs and aryl dihalides such as the Sonogashira and Glaser–Hay couplings [16,23], the classical formation of ester [12,17], amide [25–26] or imide [27] bonds between CDs or amino CDs and acid- or anhydride-linkers, and finally urea/thiourea bonds [28–29]. However, the obvious advantages of the Staudinger ligation (high reaction rates, absence of a catalyst, aqueous environment, rigid spacer/linker), have not been explored so far toward formation of CD dimers.

For any realistic application of CD oligomers, especially for drug encapsulation, some key requirements can be set: (i) ease of preparation, (ii) efficient purification, (iii) aqueous solubility of the oligomer, (iv) structural characterization regarding conformation and dynamics, intra- and intermolecular interactions, self-inclusion and aggregation in water, and ultimately (iv) availability of the individual CD cavities for molecular inclusion. The later has become of critical importance since it has been shown recently [30] that depending on the linker connecting the CD moieties, self-inclusion occurs in water, frequently associated with inversion of one glucopyranose unit, that may totally incapacitate the cavity and annihilate the prospective utility of the oligomer as a multivalent host. These important findings explain the inconsistent or unexpected behavior of CD dimers in the past [31] and call for re-evaluation of binding constants of dimers determined by various research groups.

**Scheme 2:**
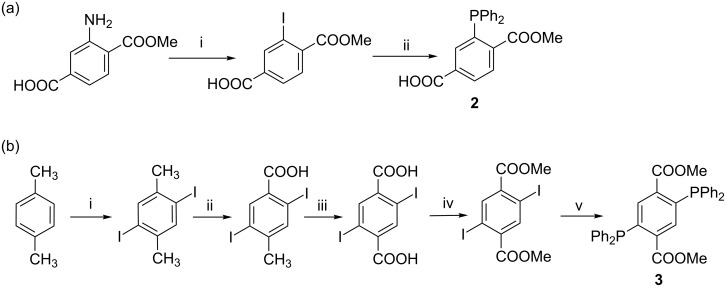
(a) i) HCl, NaNO_2_/H_2_O, then KI/H_2_O, 58%, ii) Ph_2_PH, Pd(OAc)_2_, Et_3_N, MeOH, 48%; (b) i) CH_3_COOH, H_2_SO_4_, CCl_4_, I_2_, IO_3_, 85 °C, 4 h, exclusion of light, 70%; ii) C_5_H_5_N, H_2_O, KMnO_4_, reflux, 24 h, exclusion of light, 55%; iii) KOH in water (10% w/v), KMnO_4_, reflux, 4 h, exclusion of light, 32% iv) MeOH, H_2_SO_4_, reflux, 5 h, 98% v) dry THF, dry DMF, Et_3_N, Pd(CH_3_COO)_2_, Ph_2_PH, 70 °C, 12 h, 98%.

In the present work, Staudinger ligation is used for the first time as an efficient, water compatible strategy toward CD dimer preparation. In addition to linker **2** (Scheme 2a), the new double arylphosphine methyl ester linker **3** has been developed (Scheme 2b) that enabled facile homodimer formation in a single step, in organic/aqueous medium. As an indispensable part of this approach, the full NMR spectroscopic characterization combined with theoretical calculations, revealed the full capacity of the cavities for molecular inclusion despite the dynamic equilibria between closed and open conformations of the products in water.

## Results and Discussion

Synthesis – The Staudinger ligation via linker **2** (Scheme 2) was initially explored to produce the corresponding functional monomer **4** and thus the β-CD dimer **5**. Linker **2** was prepared from methyl 2-aminoterephthalate via a sequence of diazotization–iodination–phosphanylation reactions [4] (Scheme 2a).

The new, doubly phosphanylated dimethyl terephthalate linker **3** was prepared from *p*-xylene via consequtive iodination–oxidation–esterification reactions [32–34], followed by the final phosphanylation step that afforded linker **3** in excellent yield (Scheme 2b). Compounds **2** and **3** each displayed one signal in the ^31^P NMR spectrum at −3.5 and −3.8 ppm (Supporting Information File 1, Figure S1). Compound **3** showed a single methoxy group in the ^1^H NMR spectrum (3.57 ppm) and a single carbonyl signal in the ^13^C NMR spectrum (166.8 ppm), reflecting the compound’s high molecular symmetry. Both linkers were stored under argon at −20 °C to minimize oxidation, although best results were obtained when used freshly prepared.

**Scheme 3:**
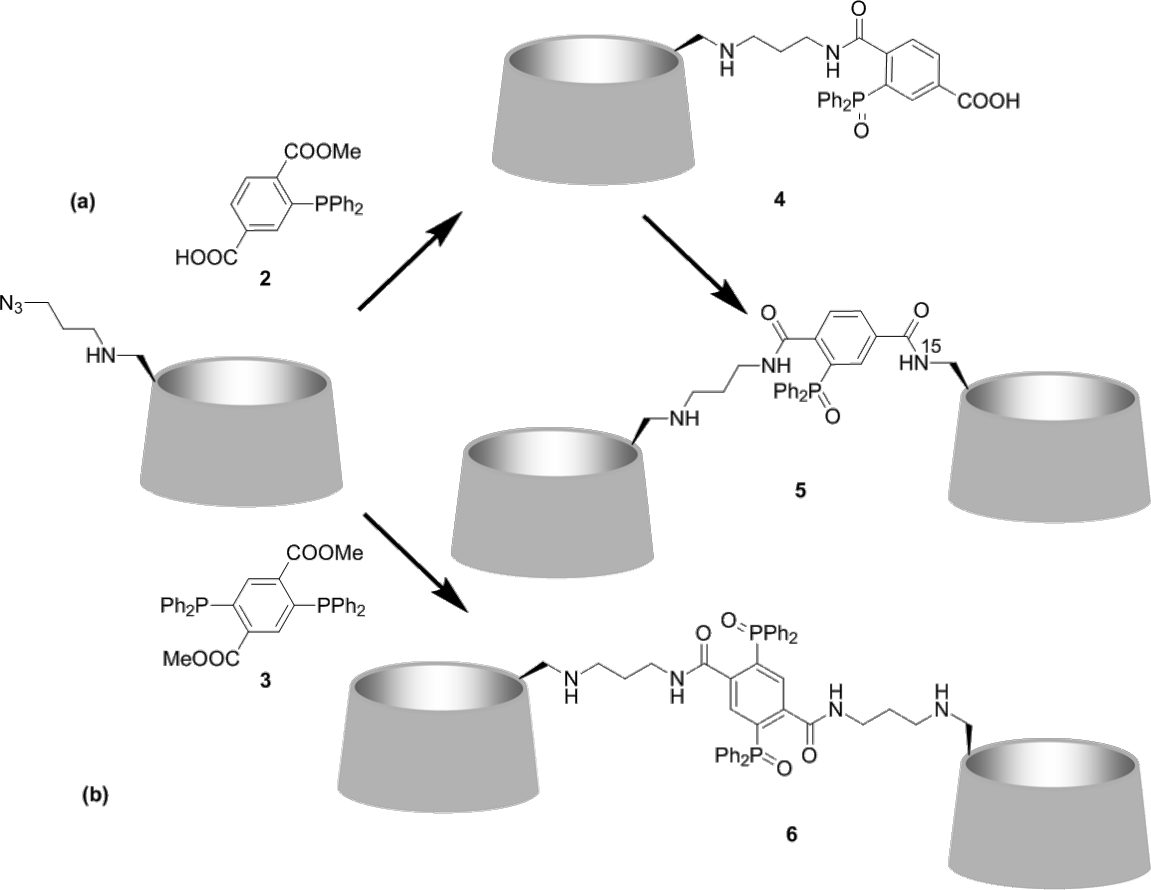
Staudinger ligation reactions: (a) Preparation of **4** from mono[6-(3-azidopropylamino)-6-deoxy]-β-CD and **2** (DMF/H_2_O, 15:1, v/v, 40 °C, 12 h, 95%) and then of dimer **5** from **4** and mono(6-^15^*N*-amino-6-deoxy)-β-CD (HATU, DIPEA, dry DMF, 10%); (b) One-step facile preparation of homodimer **6** from mono-[6-(3-azidopropylamino)-6-deoxy]-β-CD and the divalent linker **3** (DMF/CHCl_3_/H_2_O, 48 h, 62%).

Staudinger ligation using **2** and mono-[6-(3-azidopropylamino)-6-deoxy]-β-CD (Scheme 3) in dimethylformamide/water (15:1) gave monomer **4** in excellent yield (95%); in acetonitrile/water (2:1, v/v) the yield was nearly half (48%).

Bertozzi and co-workers have studied the mechanism of the Staudinger ligation of arylphosphine ester linkers such as **2** with various alkyl azide substrates [7]. They have shown that the ligation is a second order process: polar protic solvents and electron-donating substituents on the aryphosphine accelerate the reaction, while the type of the ester (methyl, aryl) does not have an effect on the rate. The actual intermediate is a five-membered phosphanazolone [7] (Scheme 1b). Double ligation to form dimer **6** was optimized taking into consideration the mechanistic aspects of the prototype reaction [7]. Thus **3** and mono-[6-(3-azidopropylamino)-6-deoxy]-β-CD were mixed in an NMR tube at 60 °C with dry CDCl_3_ and dry DMF-*d*_7_ where both reactants were completely soluble and the evolution of the ^31^P NMR signals was monitored with time. The formation of an intermediate, most likely a phosphaza ylide was evidenced by a signal at 19.8 ppm [7] that emerged following addition of the azido-β-CD in the CDCl_3_/DMF-*d*_7_ solution; however complete formation of the product required 48 h. Apparently steric factors influence the progress of the reaction, also considering the five-membered ring intermediate (Scheme 1b) required for the ligation to proceed. Addition of deuterium oxide resulted in hydrolysis of the intermediate and disappearance of the 19.8 ppm peak within ~4 h while the emergence of a peak at 35.4 ppm validated the formation of dimer **6**. The above confirmed DMF as the optimal solvent. In DMSO and CH_3_CN, numerous ^31^P signals upon dissolution of **2** or **3** were observed evidently due to secondary reactions leading to a lower yield for the ligation step.

Monomer **4** and dimer **6** displayed a single ^31^P NMR signal at 35.2 ppm and the correct mass in MALDI–TOF MS. Reaction of **4** with mono(6-^15^*N*-amino-6-deoxy)-β-CD under typical amide coupling conditions provided the desired dimer **5** (Scheme 3) but only in 10% yield. This product displayed one signal at 35.2 ppm in the ^31^P NMR spectrum and one at 141.6 ppm in the ^15^N NMR spectrum, the latter considerably deshielded compared to that of the starting mono(6-^15^*N*-amino-6-deoxy)-β-CD at 61.7 ppm, thus confirming the structure of dimer **5**. However, while the excellent yield of the Staudinger ligation step to **4** showed its applicability and suitability for cyclodextrin substrates, the poor yield of CD-dimer **5** suggested the presence of impediments in the introduction of a second β-CD moiety. Examination of the 3D structure of **4** revealed that the phenyl moieties impose steric restrictions toward the approach of mono(6-^15^*N*-amino-6-deoxy)-β-CD. The suggestion of hindrance was supported by the fact that Staudinger ligation using **2** and **3** although very successful with mono[6-(3-azidopropylamino)-6-deoxy]-β-CD, where the azido group is connected to the β-CD moiety via the flexible aminopropyl spacer, failed with mono(6-azido-6-deoxy)-β-CD. Therefore, if steric problems are circumvented using a suitable spacer between the CD macrocycle and the azido group, the process becomes very attractive: The reaction conditions are quite undemanding and mild, the purification of the products is straightforward and efficient and the yields are good to excellent. Thus the procedure could become the method of choice for a variety of CD substrates to form derivatives and either homo- or heterodimers.

Conformations of Staudinger products and guest binding in aqueous solution as derived from NMR spectroscopy. – The study of the conformations in water is essential for the evaluation of the compounds’ efficiency toward guest inclusion. The ^1^H NMR spectrum of monomer **4** and dimer **5** showed quite disperse signals (Supporting Information File 1, Figure S2), anticipated due to the lack of molecular symmetry, while the spectrum of **6** (Figure S2) was simpler reflecting a rather symmetrical structure. The 2D ROESY NMR spectra of **4** and **5** suggested formation of self-inclusion complexes via the phenyl groups of the linker, since strong dipolar interactions between the narrow side protons (H5, H6,6’, dispersed over a wide range of frequencies) and the phenyl groups were observed (the terephthalate moiety did not participate) (Figure S2). Self-inclusion could arise intramolecularly by rotation of the aminopropyl spacers in order to effect insertion of the phenyl groups in the narrow side of the cavity, but also intermolecularly, as indirectly evidenced by the strong concentration dependence of the chemical shifts in the ^1^H NMR spectrum in D_2_O. Likewise, the 2D ROESY spectrum of **6** in D_2_O (Supporting Information File 1, Figure S3) showed clearly that the phenyl groups developed strong through-space interactions with the cavity protons H3 (all signals bundled together in an apparent quartet) and weaker with H5, and H6,6’ (signals grouped together, as well). The indicated self-inclusion could take place intermoleculary from the wider β-CD side as well as intramolecularly from the narrow side. Such self-inclusion has been observed and taken advantage for selective catalysis in recently published cyclodextrin-phosphanes [35–37]. Moreover, some of the observed interactions could arise from the proximity of the phenyl and β-CD moieties in the dimeric structure. In order to improve our understanding and separate the water-induced inclusion configurations from the ones imposed by the bulkiness of the molecules, the 2D ROESY spectra of **4** and **6** in DMSO-*d*_6_ were examined. The spectra revealed that the phenyl protons indeed develop ROE interactions with the primary side β-CD protons H5 and H6.H6’, as well as with all the aminopropyl chain protons in monomer **4** (Figure S4). The above suggest that the phenyl groups of the linker prefer to linger over the narrow β-CD opening. Likewise, dimer **6** in DMSO-*d*_6_ (Supporting Information File 1, Figure S5) displayed through space interactions between the phenyl protons and the H1, H2, H4 external to the β-CD cavity as well as the primary side H5, H6,6’ and OH6 (and not with the secondary side OH2, OH3), indicating that in **6** (much more than in **4**) the phenyl moieties are located over the narrow opening of the β-CD moiety at close distance. In both **4** and **6**, dipolar interactions with cavity proton H3 did not seem to develop, as indicated by the assignments of the signals, thus ruling out the self-inclusion in DMSO. The above show that configurations with phenyl groups over the narrow β-CD opening, as observed in DMSO, evolve in two limiting ways in water: either open up entirely exposing the phenyl moieties to a neighboring cavity of another molecule, or close in by self-inclusion in their own cavity.

In order to evaluate the extent of intermolecular interactions in dimer **6** in D_2_O, 2D diffusion ordered spectroscopy (DOSY) was used. The diffusion coefficient *D*_6_, of **6** (1 mM, Supporting Information File 1, Figure S6), was found 1.7 × 10^−11^ m^2^/s while in the presence of four equivalents of 1-adamantylamine hydrochloride (**ada**) it increased to *D*_(6/ada) =_ 2.4 × 10^−11^ m^2^/s, suggesting a faster motion of the complex **6**–**ada** in the solution, presumably due to the breaking apart of intermolecularly associated dimers. Comparing the above values with those of natural β-CD (0.5 mM [38], *D*_β-CD_ = 3.29 ± 0.07 × 10^−10^ m^2^/s) a ratio of *D*_β-CD/_*D*_6_ ≈ 19 is obtained, much larger than the corresponding molecular weight ratio, FW_β-CD_/FW_6_ ≈ 0.38, while *D*_(6/ada)_/ *D*_6_ ≈ 1.3, revealing the important effect of intermolecular attractive forces on the translational motion of the dimer. The inclusion of **ada** evidently helped to reduce these forces but aggregation was not totally prevented. It is known that β-CD forms aggregates in aqueous solution as shown by DLS [39] and DOSY measurements at different concentrations [38,40–41]; it is therefore reasonable to assume that a part of the aggregation of the dimer is due to the β-CD moieties. Finally, given that in any host–guest solution in the fast exchange regime in the NMR time scale,

*D*_obs_ = *D*_bound_ *f*_bound_ + *D*_free_ (1 − *f*_bound_) [42] (*f* = mole fraction)

the observed *D*_ada_ ≈ 6.6·10^−11^ m^2^/s, suggests that the guest diffuses at a rate close to that of free **ada**, because (i) there is an excess of it in the solution deliberately added to maximize the complex concentration and (ii) the binding constant is moderately strong, therefore an average diffusion coefficient is observed.

**Figure 1:**
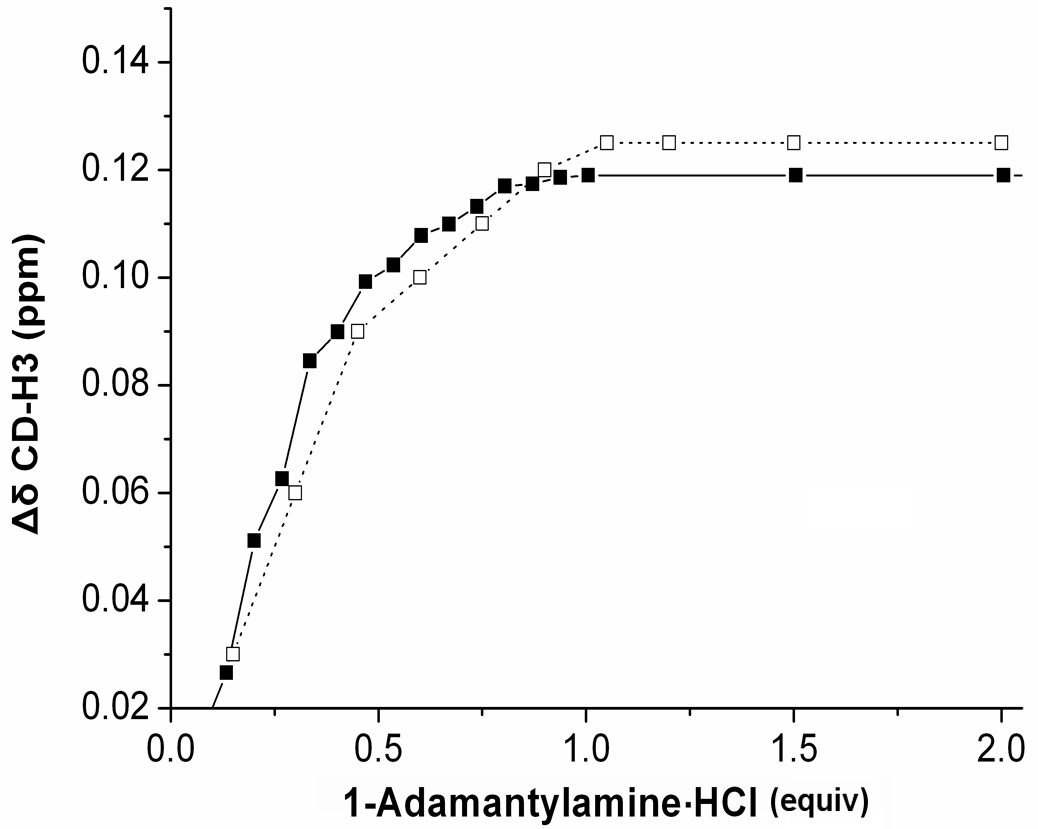
^1^H NMR chemical shift change (Δδ) of CD cavity Η3 signal of compounds titrated with 1-adamantylamine·HCl (**ada**) in D_2_O (500 MHz, 298 K): a) monomer **4** (1 mM, filled squares, solid line) and b) dimer **6** (1 mM) (empty squares, dotted line) plotted per cavity vs ½ concentrations of **ada**.

Cavity availability for inclusion complexation. – The above results suggest that the phenyl groups might be serious competitors to any incoming guest molecule. To test the accessibility of the cavities and the usability of the products as molecular carriers, titration of **4** and **6** with **ada** in D_2_O were carried out. The corresponding plots (Figure 1) revealed that one equivalent of **ada** for monomer **4** and two equivalents of **ada** for dimer **6** were required to saturate the chemical shifts of the cavity proton, H3, suggesting full binding capacity of all available cavities**.** When the chemical shift changes of H3 [Δδ(CD-Η3] of **6** were plotted vs ½ concentration of the titrant (i.e. per cavity) (Figure 1), the resulting curve nearly coincided with that of the curve of monomer **4**. The striking similarity of the induced chemical shift displacements of CD-Η3 for **4** and **6**, reveal a similar mode of inclusion, simultaneous and independent for each cavity of **6** and subsequently very similar association constants. Indeed, non-linear fitting of the observed shifts to a suitable equation for 1:1 binding in the fast exchange regime [43] showed that the association constants, as log*K,* are in the order of ~4.2 i.e. comparable with those reported for the binding of β-CD alone with the same guest [44]. Therefore the strength of the binding did not reveal severe competition from the phenyl groups of the spacers.

The through-space interactions between the phenyl protons and those of β-CD in **6** and **4** (Supporting Information File 1, Figure S3) clearly changed in the presence of **ada** (Supporting Information File 1, Figure S7). Specifically, spatial proximity of phenyl groups with H6 (and not β-CD-H3, as clearly assigned from the HSQC spectra) and H5, H6’, is evident from the 2D ROESY spectra, suggesting that upon entrance of **ada** from the wider side, the phenyl moieties are lifted over the narrow opening. In both molecules **ada** is inserted with the amino group protruding from the wider β-CD side (Figure S7).

In summary, NMR experiments have shown that the compounds, although they form intra- and intermolecular complexes in aqueous solution by self-inclusion, are accessible by external guest molecules while the strength of binding does not seem to decrease by the presence of the linker moieties, compared to the parent β-CD. In addition to closed configurations of **4** and **6** in water formed by intramolecular phenyl inclusion, open configurations are additionally present, which promote intermolecular aggregation and can account for the slow Brownian motion of the dimer in the aqueous solution. The fact that in DMSO the linkers prefer to reside over the cavity indicates that intermediate conformations, between extended and self-included may exist in water as well.

## Computational results

**Figure 2:**
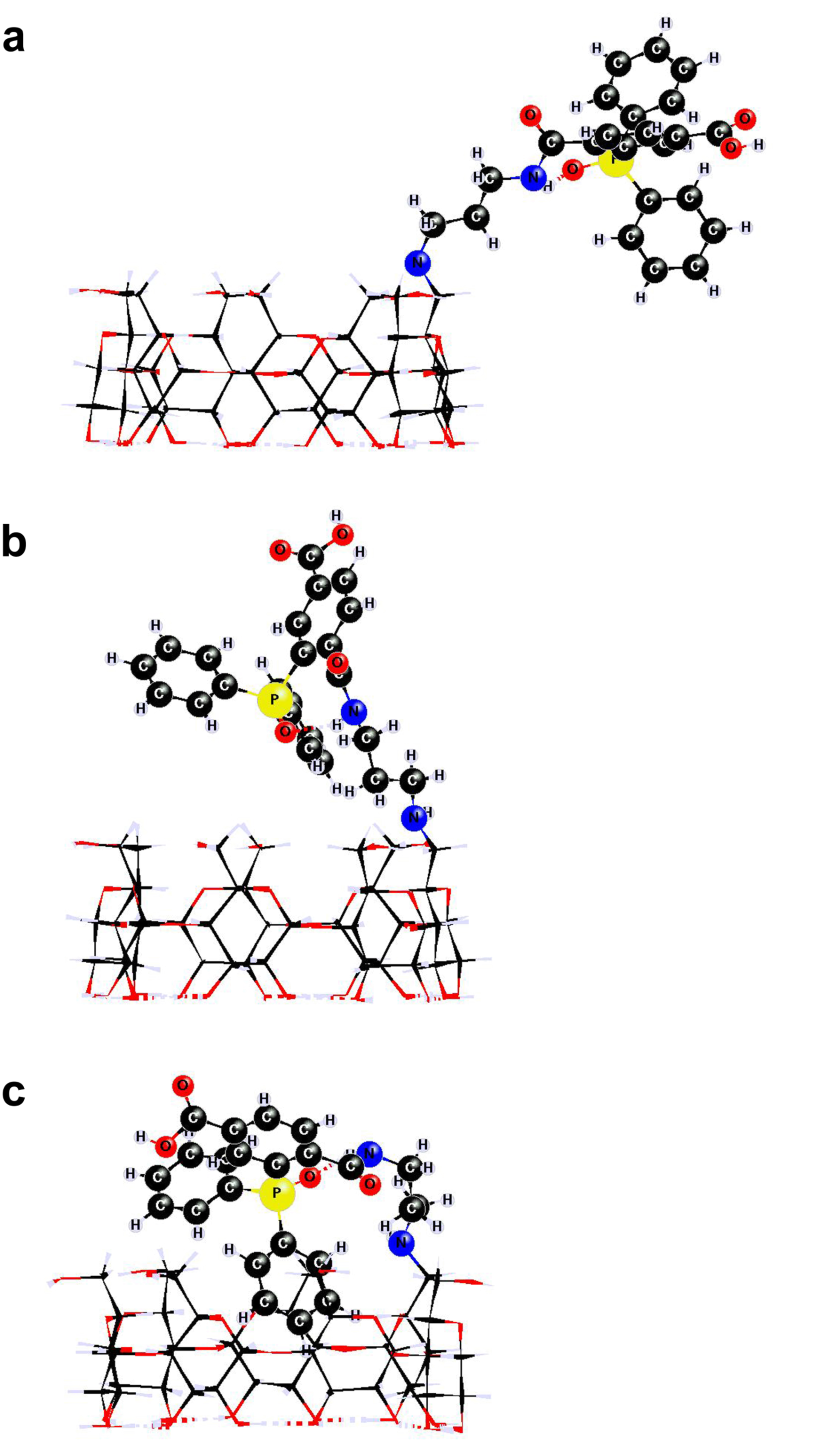
The most stable conformations of **4** at the PM3(COSMO) level of theory: (a) open, (b) vicinal, and (c) inclusion conformation.

**Figure 3:**
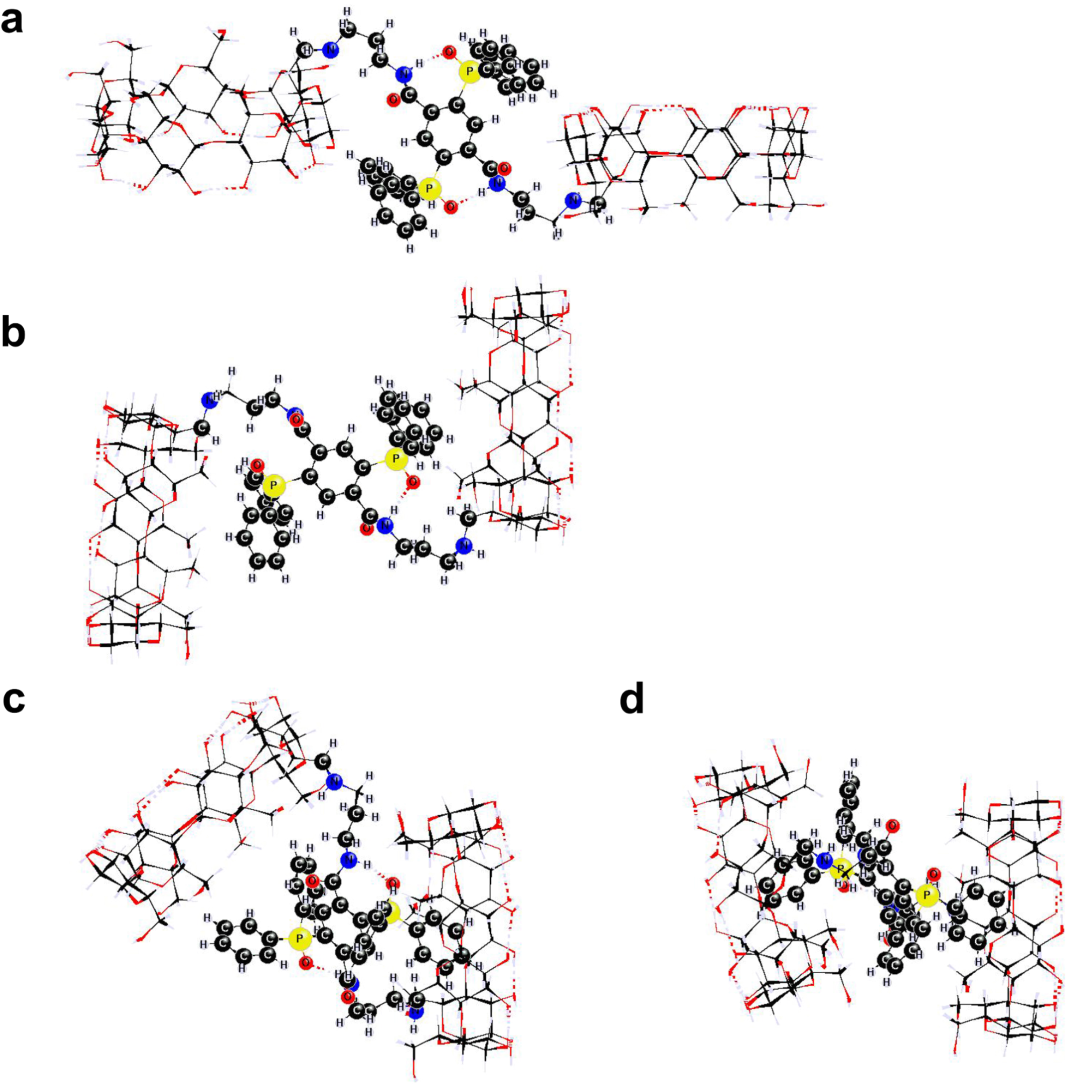
Typical conformations of **6**: (a) open conformation, (b) vicinal, (c) inclusion/vicinal and (d) double inclusion conformation.

Quantum mechanical calculations were carried out for monomer **4** and dimer **6** at the PM3 level of theory for isolated molecules, as well as in the presence of solvent (water) at the PM3(COSMO) level of theory in order to assess solvation effects. For critical configurations of **4**, the PM3 results were compared against those calculated by DFT at the B3P86/6-31G(d',p') level of theory. The average structural deviation of the PM3 geometries from those derived at the B3P86/6-31G(d',p') level was reasonably small, 0.015 Å for bond lengths, whereas for bond and dihedral angles the average deviation was 2.1 and 7.2 degrees, respectively. Several initial geometries with a varying degree of phenyl groups’ orientation and proximity to β-CD (stemming mainly from the torsional flexibility of the aminopropylamino–spacer moiety) were fully optimized at the PM3(COSMO) and PM3 level of theory. The calculated geometries were sorted out into three limiting configurations: i) open, in which the phenyl rings are positioned on the exterior of β-CD, ii) vicinal, in which two phenyl rings are close to the primary side rim of β-CD and iii) inclusion, in which one phenyl ring penetrates inside the β-CD cavity. A *gauche*–*trans* (*gt*) arrangement of the C5-C6OH moieties in **4** was found to disfavor the inclusion configuration by more than 10 kJ/mol, due to the subsequent contraction of the primary entrance of the β-CD cavity, compared to a *gauche–gauche* (*gg*) arrangement. The energies of PM3(COSMO) as well as PM3 for isolated molecules, for the various configurations of **4** (Figure 2) span a range of 25 kJ/mol, with the inclusion case being the most thermochemically favorable, closely followed by the vicinal case. The PM3(COSMO) energies of the corresponding configurations for dimer **6** (Figure 3) span a greater range, 65 kJ/mol, with a mixed inclusion/vicinal configuration (Figure 3c) being the most thermochemically stable. However, configurations with phenyl groups immersed in both β-CD cavities (Figure 3d) are the least stable by PM3(COSMO) (in the presence of water), although highly favorable by PM3 (in the absence of solvent). This was attributed to insufficient hydration of the primary hydroxy groups due to steric crowding around the primary sides of the two β-CD tori. The DFT energies for critical configurations of **4** span a range of 17 kJ/mol, comparable to the range of PM3 energies. Thus, semiempirical as well as DFT calculations suggest that both compounds prefer to adopt conformations with the phenyl groups either inside the CD cavity (inclusion) or over the C6 area (vicinal) of comparable energies, therefore they may easily interconvert.

The energetics of intermolecular inclusion of a phenyl group inside the β-CD cavity for a pair of monomers **4** having the open configuration was explored at the PM3(COSMO) level of theory. A variety of bimolecular arrangements was considered, by also taking into account the possibility of phenyl inclusion in either side of β-CD. For an all-*gg* conformation of C5-C6OH in **4**, phenyl inclusion via the primary side was more thermochemically favored by ca. 40 kJ/mol, whereas for an all-*gt* conformation, inclusion via the secondary side was more favored by ca. 45 kJ/mol, attributed to the contraction of the primary side opening. The geometries for the most stable arrangements for a pair of **4**, are shown in Supporting Information File 1, Figure S8.

The shortest distances between hydrogen atoms of the closest phenyl ring and each kind of glucose hydrogen atom of the β-CD for the most stable configuration of each limiting case are shown in Supporting Information File 1, Table S1. In the inclusion structures of **4** and **6** the calculated shortest distances of the inserted phenyl moiety were found only with the β-CD cavity H3, H5 and H6,6'. In the vicinal lowest-energy configurations, the corresponding calculated shortest distances of both phenyl groups were found only with the primary side protons H5 and H6,6’ and not with H3, in agreement with NMR data in DMSO. Finally, in the open structures the shortest distances observed were with the cavity exterior H1, H2 and H4. The above are in line with the experimental findings in the 2D ROESY NMR data in D_2_O. Intermolecular arrangements (Supporting Information File 1, Figure S8), verified by the experimentally observed aggregation, may also incorporate phenyl group inclusion complexes with similarly short distances between phenyl hydrogens with H3, H5 and H6,6', shown in Table S1 (Supporting Information File 1) and in line with the NMR data.

The accommodation of the protonated form of 1-adamantylmine inside the cavity of monomer **4** was also explored at the PM3(COSMO) level of theory. A variety of initial complex geometries was considered consisting of vicinal as well as of inclusion configurations for **4**. The resultant optimized geometries reveal that **ada** is encapsulated inside the β-CD cavity by its hydrophobic alkyl side leaving the protonated amine moiety well outside the cavity. Its accommodation into the inclusion configuration proceeds by a push of the phenyl ring. Moreover, the PM3(COSMO) level of theory suggests that **ada** accommodation by **4** is energetically favorable by ca. 45 kJ/mol. The geometries of two typical complexes of **4** with **ada** are shown in Supporting Information File 1, Figure S9.

## Conclusion

The present work demonstrates the application of the Staudinger ligation reaction for the first time to form β-CD dimers in good yields under mild conditions in aqueous/organic media. A new double Staudinger linker has been specifically developed to allow homodimer formation in one step. Despite the well verified formation of intra- as well as intermolecular inclusion complexes, the compounds proved to be effective in encapsulating a suitable external guest in each cavity. The data were fully supported by theoretical calculations that confirmed the energetic preference for the self-inclusion configurations. The method can be clearly utilized for dimer formation using other cyclodextrin azides, provided that there is a long enough spacer connecting the azido group with the CD macrocycle.

## Supporting Information

The Supporting Information provides full experimental procedures and detailed analytical data for the synthesis of all compounds; additional 2D NMR spectra; the computational procedure and a table of intramolecular distances for the three limiting conformations of **4** and **6** and for a pair of **4**. Theoretical geometries of intermolecular dimers of **4** and of the complex **4**/**ada** are also shown.

File 1 Experimental, analytical and computational data.
