# Supplementary material for: Staudinger ligation towards cyclodextrin dimers in aqueous/organic media. Synthesis, conformations and guest-encapsulation ability
Source: Beilstein J Org Chem. 2014 Apr 3;10:774–83. doi: 10.3762/bjoc.10.73 (PMC3999877; doi:10.3762/bjoc.10.73)
Supplement: File 1 — Experimental, analytical and computational data. [file Beilstein_J_Org_Chem-10-774-s001.pdf]

Supporting Information

for

Staudinger ligation towards cyclodextrin dimers in aqueous/organic media. Synthesis, conformations and guest-encapsulation ability

Malamatenia D. Manouilidou, Yannis G. Lazarou, Irene M. Mavridis, Konstantina Yannakopoulou*

Address: Institute of Advanced Materials, Physicochemical Processes, Nanotechnology & Microsystems, National Center for Scientific Research "Demokritos", Terma Patriarchou Gregoriou & Neapoleos, Aghia Paraskevi Attikis, 15310 Greece. Tel. +30210 6503796, Fax: +30 210 6511766

Email: Konstantina Yannakopoulou - dyanna@chem.demokritos.gr

*Corresponding author

Experimental, analytical and computational data

Experimental

NMR experiments were performed on either a 250 MHz Avance III or a 500 MHz Avance DRX Bruker NMR spectrometer using the library 2D sequences and 90 degree pulses. For ROESY experiments, a spinlock time of 300–350 ms was used. DOSY NMR experiments: Samples of 4 cm height were used to ensure gradient linearity along the volume. The temperature was kept at 298.0 ± 0.1 K (air flow 535 L/min). The measured 90 degree pulses were 10 μs. A delay d1 = 4*T_1 was inserted for the measurements, which were carried out with the gradient spin echo sequence *ledbpgspr2s1d* employing a presaturation step. Data were acquired with 32 or 16 scans for each gradient step, 4 dummy scans, a 2.5 ms gradient pulse (δ), a linear gradient of 16−32 steps between 2% and 95% and diffusion time (Δ) 100 ms. Total acquisition times were 31 min or 62 min. Processing was carried out with Bruker's Topspin 2.1 software

Synthetic procedures

1,4-Diiodo-p-xylene, 4-methyl-2,5-diiodobenzoic acid [1], 2,5-diiodoterephthalic acid [1], dimethyl 2,5-diiodoterephthalate [2] and mono(6-p-toluenesulfonyl)-β-cyclodextrin [3] were prepared according to the literature. Linker **2** was prepared according to ref. [4].

*Dimethyl 2,5-bis(diphenylphospanyl)terephthalate (**3**).–* To a solution of dimethyl 2,5-diiodoterephthalate [2] (100 mg, 0.224 mmol) in dry THF (3 mL), triethylamine (125 μL, 0.896 mmol) was added followed by a catalytic amount of palladium acetate. The solution was degassed using argon, diphenylphosphine was added (78 μL, 0.448 mmol) and the solution was left under argon and stirring at 70 °C for 12 h. Then, the solution was allowed to acquire rt and the solvent was evaporated under reduced pressure. The residue was dissolved in $CHCl_3$:H_2O (1:1, v/v) (84 mL), the two phases were separated and the organic phase was washed with $HCl_{(aq)}$ (1 M, 4 mL). Then, the solvent was evaporated under reduced pressure

and a small amount of DMF was added to the resulted solid. The desired product was isolated after centrifugation as an orange solid and dried under vacuum (126 mg, 100%). The product was stored under argon at −20 °C. R_f = 0.57 (eluent: $CHCl_3$). m.p.: 199.0-201.0 °C (dec). ^{1}H NMR ($CDCl_3$, 500 MHz, 298 K) δ 7.55-7.40 (m, 22H, -Ar), 3.57 (s, 6H, $-OCH_3$). ^{13}C NMR ($CDCl_3$, 125 MHz) δ 166.8 (-COO-), 141.1, 140.9 (C1), 137.1 136.9, 136.1, 134.2, 134.0, 129.2, 128.8 (-Ar), 52.2 ($-OCH_3$). ^{31}P NMR ($CDCl_3$, 202.5 MHz, 298K) δ -3.9. MS (ESI): m/z 563.3 $[M+H]^+$, calcd. for $[C_{34}H_{27}O_4P_2H]^+$: 563.15.

Mono[6-(3-azidopropylamino-6-deoxy)]-β-cyclodextrin. *A* solution of dry mono(6-p-toluenesulfonyl)-β-cyclodextrin [3] (80 mg, 0.06 mmol) in 3-azidopropylamine (0.1 mL) was left under stirring for 14 h at 80 °C. H_2O then was added (5 mL) and the solvents were evaporated under reduced pressure. The oily mixture was dissolved in H_2O, the pH of the solution was adjusted to 9 with $NaOH_{(aq)}$ (10%, v/v) and a cation-exchange resin (Amberlite IR120, H^+, 1.4 g) was added to remove extra 3-azidopropylamine. The mixture was filtered, the pH of the filtrate was adjusted to 9 with $NaOH_{(aq)}$ (10%, v/v) and a anion-exchange resin (Amberlite IRA-400, 600 mg) was added to remove free *p*-toluenesulfonic acid. The mixture was filtered, the pH of the filtrate was adjusted to 7 with $HCl_{(aq)}$ (10%, v/v) and it was dialyzed for 3 h to remove the salts. After evaporating the solvents under reduced pressure, the product was isolated as a white powder, (483 mg, 85%). R_f = 0.25 (eluent *i*-PrOH:AcOEt:H_2O 5:3:1). ^{1}H NMR (D_2O, 500 MHz, 298 K) δ 5.31 (bs, 2H, H1), 5.03 (bs, 5H, H1), 4.11-3.65 (26H, H3, H5, H6), 3.65-3.47 (14H, H2, H4), 3.4 (bs, 2H, H7), 3.06 (d, J = 13.0 Hz, 1H, $H6_A$), 2.78 (dd, J = 13.0 Hz, J = 4.0 Hz, 1H, $H6'_A$), 2.69 (m, 2H, H9), 1.80 (m, 2H, H8). ^{13}C NMR (D_2O, 298 K, 125 MHz) δ 102.4, 100.7 (C1), 81.4 (C4), 75.9 (C3), 72.7 (C2), 71.9 (C5), 60.3 (C6), 49.3 ($C6_A$, C7), 45.6 (C9), 27.9 (C8). IR (cm^{-1}) ν 2103.6 ($-N_3$). MS (MALDI-TOF): m/z 1218.0 $[M + H]^+$, calc. for $[C_{45}H_{76}N_4O_{34}H]^+$: 1218.09.

Monomer **4**. To a solution of dry mono-[6-(3-azidopropylamino)-6-deoxy]-β-cyclodextrin (30 mg, 0.024 mmol) in dry DMF (1.5 mL), 1-methyl-2-diphenylphosphanylterephthalate [4] (11.37 mg, 0.03 mmol) was added during 1 h and the solution was left under stirring for 15 min at 40 °C. Then, H_2O (0.1 mL) was added and the solution was allowed to stir for 12 h at 40 °C. The solvents were evaporated under reduced pressure and the solid produced was dissolved in H_2O and was washed with $CHCl_3$ (4 × 15 mL) at pH 2. The aqueous phase was isolated, its pH was adjusted at 7 and was subjected to solid-phase extraction (SPE, Alumina-A) with H_2O as the eluent. The desired product was isolated as white powder (35.1 mg, 95%). R_f = 0.29 (eluent: *i*-PrOH:AcOEt:H_2O 5:3:1). ^1H NMR (D_2O, 500 MHz, 298 K) δ 8.27 (d, *J* = 14.0 Hz, 1H, H2), 8.23 (d, *J* = 8.0 Hz, 1H, H6), 7.86-7.69 (m, 5H, 4xH3′+ H5), 7.70-7.60 (m, 5H, 2xH1′, 4xH2′), 5.18-4.98 (m, 7H, H1), 4.10-3.72 (m, 21H, H3, H5, H6), 3.73-3.51 (m, 18H, H2, H4, H6), 3.47 (t, *J* = 9.0 Hz, 1H, H4), 3.13 (d, *J* = 11.0 Hz, 1H, H6), 2.96 (m, 1H, Hc), 2.94-2.79 (m, 2H, Hc′& H6′), 2.68 (m, 2H, Ha), 1.69 (m, 2H, Hb). ^{13}C NMR (D_2O, 62.9 MHz) δ 173.1 (COOH), 171.0 (HN-C=O), terephthalic 141.8 (C1), 138.7 (C4), 134.7 (C2), 133.5 (C6), 133.4-126.9 (Ph, C1′, C2′, C3′, terephthalic C5), 102.0 and 101.8 (CD-C1), 83.6, 81.9-80.1 (all CD-C4), 73.5-71.22, 70.24 (CD C2, C3, C5), 60.6-59.1 (CD-C6), 49.7 (CD-$C6_A$), 46.0 (chain Cc), 36.7 (chain Ca), 27.5 (chain Cb). ^{31}P NMR, (D_2O, 202.5 MHz, 298K) δ 35.2. MS (MALDI-TOF): m/z 1539.1 $[M+H]^+$, 1561.1 $[M+Na]^+$ and 1577.1 $[M+K]^+$, calcd. for $[C_{65}H_{91}N_2O_{38}PH]^+$: 1539.5, $C_{65}H_{91}N_2O_{38}PNa^+$: 1561.5, $C_{65}H_{91}N_2O_{38}PK^+$: 1577.5.

Dimer **5**. To a solution of monomer **4** (48 mg, 0.03 mmol) in dry DMF (2 mL), HATU (1-[bis(dimethylamino)methylene]-1*H*-1,2,3-triazolo[4,5-b]pyridinium 3-oxide hexafluoro-phosphate, Aldrich] was gradually added (947.2 mg, 0.12 mmol) and the solution was left under stirring for 1 h at 0 °C. Diisopropylethylamine (15.8 μL, 0.09 mmol) was subsequently added at rt until the pH of the solution was 8–9. Finally, mono(6-^{15}N-amino-6-deoxy)-β-cyclodextrin (38.7 mg, 0.03 mmol) was gradually added and the solution was stirred for 72 h

at 35 °C. During the reaction, the pH was always adjusted to 9 with additional diisopropylethylamine. The product was purified by successive solid-phase extractions (an SPE column packed with Alumina-A followed by an SPE column packed with silica gel modified with carboxy groups), by which excessive mono(6-^{15}N-amino-6-deoxy)-β-cyclodextrin was removed. The pH of the solution was adjusted to 7 and dialyzed for 12 h. The desired product was isolated as a white solid (4 mg, 10%). R_f = 0.06 (eluent *i*-PrOH:AcOEt:H_2O 5:3:1). ^{1}H NMR (D_2O, 298 K, 500 MHz) δ 8.19 (d, *J* = 8.0 Hz, 1H, terephthalic H6), 8.05-7.37 (m, 12H, Ar), 5.23-5.00 (m, 14H, CD-H1, all), 4.18-3.85 (m, 53H, H3, H5, CD1-H6, CD2-H6) 3.84-3.33 (m, 31H, H2, H4, 2xH6′, H6′), 3.28 (bs, 1H, CD2-H6′), 3.02 (bs, 3H, chain Hc, Ha), 1.89 (bs, 2H, chain Hb). ^{13}C NMR (D_2O, 298 K, 62.9 MHz) δ 139.0-124.9 (Ar), 101.8 (CD-C1), 80.9, 80.6, 80.1 (all CD-C4), 73.0, 72.0, (CD C2, C3, C5), 59.9, 59.6 (CD1-C6), 48.8 (CD2-C6), 45.4 (chain Cc), 41.1 (CD2-C6), 35.8 (chain Ca), 25.7 (chain Cb). ^{31}P NMR (D_2O, 298K, 202.5 MHz) δ 35.2. ^{15}N NMR (D_2O, 298K, 50.7 MHz) 141.6. MS (MALDI-TOF): m/z 2658.66 $[M+H]^+$ and 2680.56 $[M+Na]^+$, calcd. for $[C_{119}H_{169}N_3O_{71}P_2H]^+$: 2656.37, $[C_{119}H_{169}N_3O_{71}P_2Na]^+$: 2678.37.

Dimer ***6.*** To a solution of mono[6-(3-azidopropylamino)-6-deoxy]-β-cyclodextrin (12.5 mg, 0.01 mmol) in DMF (450 μL), dimethyl 2,5-(diphenylphosphanyl)terephthalate was added as a solution in dry $CHCl_3$ (2.7 mg, 0.0047 mmol in 100 μL of dry $CHCl_3$). The reaction was stirred for 48 h, under argon, at 60 °C. H_2O was then added (20 μL) and the reaction was left under stirring at 60 °C for additional 4 h. The reaction was terminated by addition of cold water and the solvents were evaporated under reduced pressure. The product was purified on a silica gel column chromatography using CH_3CN:H_2O (5:2 to 1:1, v/v) and then EtOH:H_2O (1:1, v/v) as eluting solvents. The pure product was isolated as a white powder (8.4 mg, 62%). R_f = 0.08 (eluent: CH_3CN:H_2O 5:2). ^{1}H NMR (D_2O, 298 K, 500 MHz) δ 7.94-7.44 (m, 22H, 20xH-Ph, H2, H5), 5.06 (s, 14H, H1), 3.96 (br apparent d, *J* = 9.0 Hz, 14H, H3), 3.90-3.70

(m, 38H, H5 & H6), 3.69-3.53 (m, 36H, H2 & H4), 3.44 (t, J = 9.0 Hz, 2H, H4), 2.92 (apparent d, J = 10.0 Hz, 2H, H6), 2.83 (s, 4H, chain Hc), 2.71 (t, J = 9.0 Hz, 2H, $H6_A$), 2.41 (s, 4H, chain Ha), 1.49 (s, 4H, chain Hb). ^{1}H NMR (DMSO-d_6, 298 K, 500 MHz) δ 8.45 (s, 2H, -NH-C=O), 7.79-7.71 (dd, J = 13.0 Hz, J = 3.0 Hz, 2H, H2), 7.70-7.57 & 7.56-7.44 (20H, H-Ph), 5.88-5.60 (m, 28H, OH2 & OH3), 4.82 (s, 14H, H1), 4.62-4.34 (m, 12H, OH6), 3.76-3.45 (m, 52H, H3, H5, H6), 3.44-3.30 (m, 24H, H2, H4), 2.78 (d, J = 10.0 Hz, 2H, $H6_A$), 2.74-2.58 (m, 6H, 2x$H6_A$, 4xHc), 2.36 (bs, 2H, Ha), 2.30 (bs, 2H, chain Ha), 1.27 (t, J = 6.0 Hz, 4H, chain Hb), 1.23 (s, 2H, -NH-). ^{13}C NMR (D_2O, 125 MHz) δ 168.6 (HN-C=O), 141.5 (Ph, C3), 135.0-127.8 (Ph, C1, C2, C4), 102.0, 101.7 (CD-C1), 83.6, 81.0 (CD-C4), 73.2 (CD-C3), 72.2 (CD-C5), 72.0 (CD-C2), 60.1 (CD-C6), 49.5 (CD-$C6_A$), 46.1 (chain Ca), 37.2 (chain Cc), 27.8 (chain Cb). ^{31}P NMR (D_2O, 298K, 202.5 MHz) δ 35.4. MS (MALDI-TOF): m/z 2912.1 $[M+H]^+$, 2934.6 $[M+Na]^+$, calcd. for $[C_{122}H_{176}N_4O_{71}P_2H]^+$: 2912.0, $[C_{122}H_{176}N_4O_{71}P_2Na]^+$: 2934.0.

Computational procedure

All semiempirical calculations were performed by the MOPAC 2012 program (Version 13.071L),[5] using the PM3 parameterization [6]. Molecular geometries were fully optimized by the Eigenvector Following approach (EF), using convergence criteria corresponding to a maximum gradient norm of 0.05 kcal mol^{-1} Å^{-1}. Solvation effects were treated by the Conductor-like Screening Model (COSMO) approach [7], using a dielectric constant of 78.4 (at 298 K)[8] and a radius of 1.3 Å for water. Density functional theory calculations were performed by the Gaussian 03 suite [9], using the B3P86 functional [10] with the 6-31G(d',p') basis set [11].

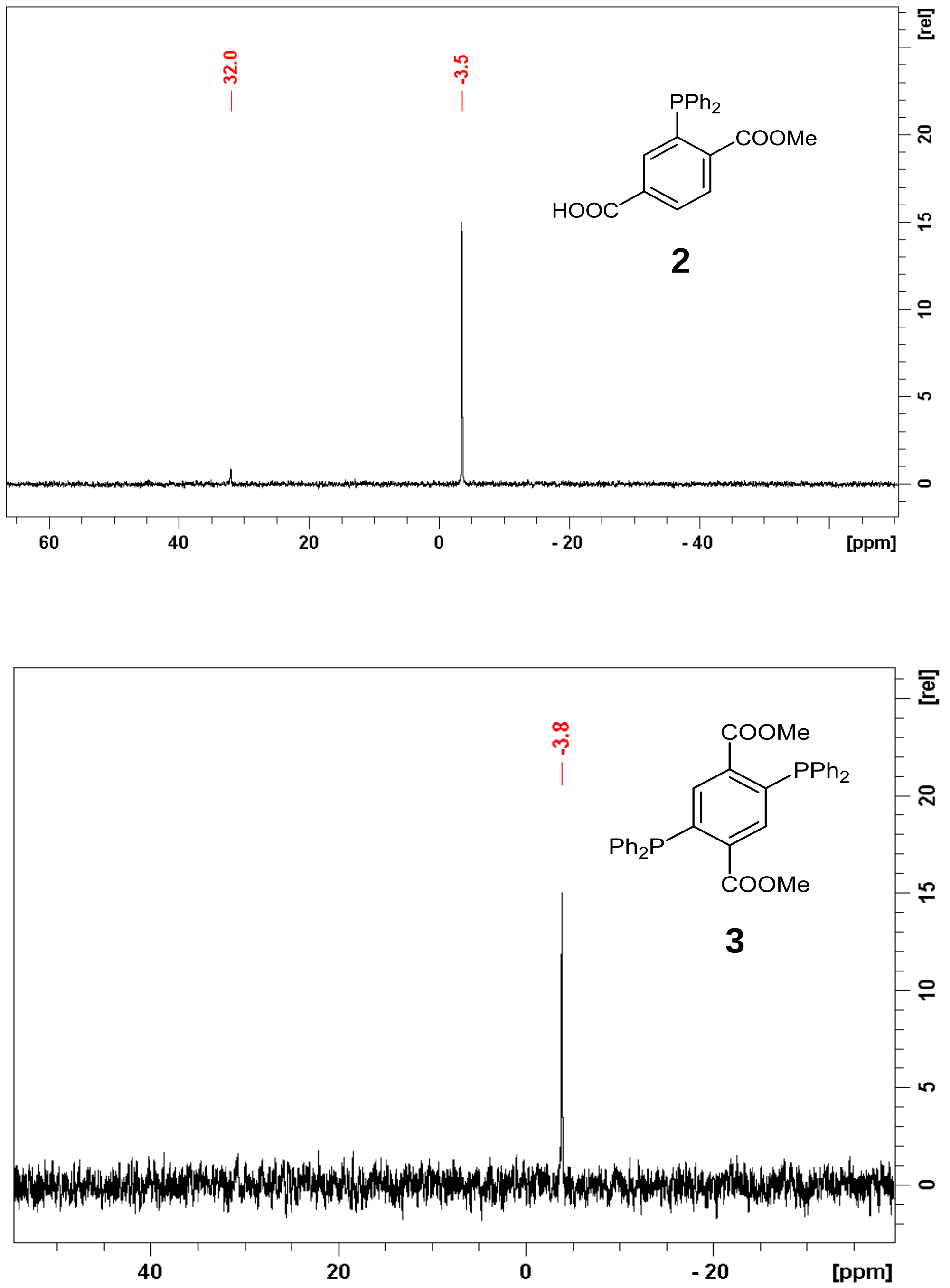

Figure S1: ^{31}P NMR spectra of linkers **2** and **3** in dry CDCl3 (202.46 MHz, 298 K).

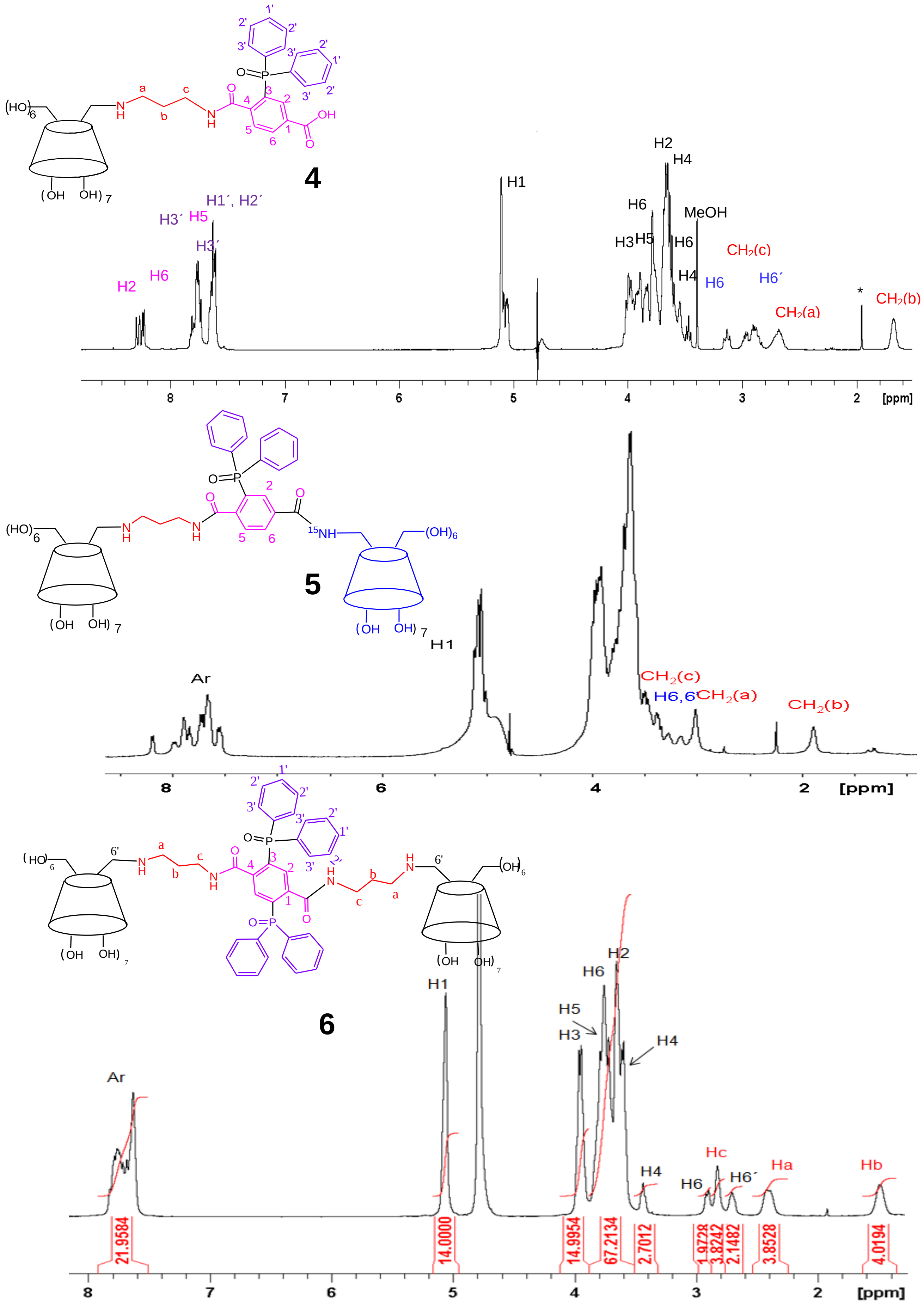

Figure S2: ^{1}H NMR spectrum of Staudinger products in D_2O (500 MHz, 298 K): of **4** and **5** with presaturation of HDO and of **6**.

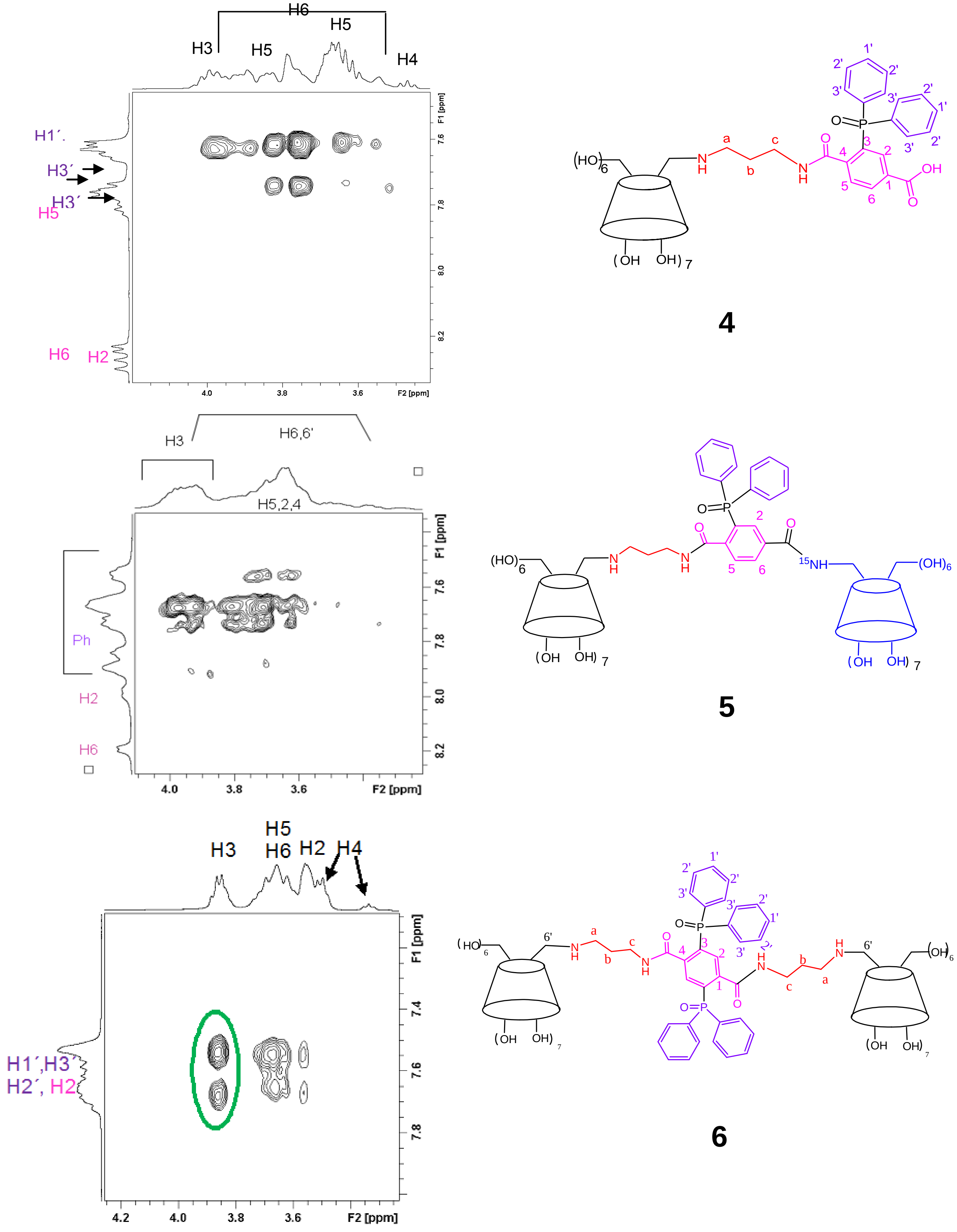

Figure S3: Partial 2D ROESY NMR spectra of monomer **4** and of dimers **5** and **6** in D_2O (500 MHz, 298K): interactions between phenyl (and not terephthalic) protons and all βCD cavity protons (H3, H5, H6,6’) are observed.

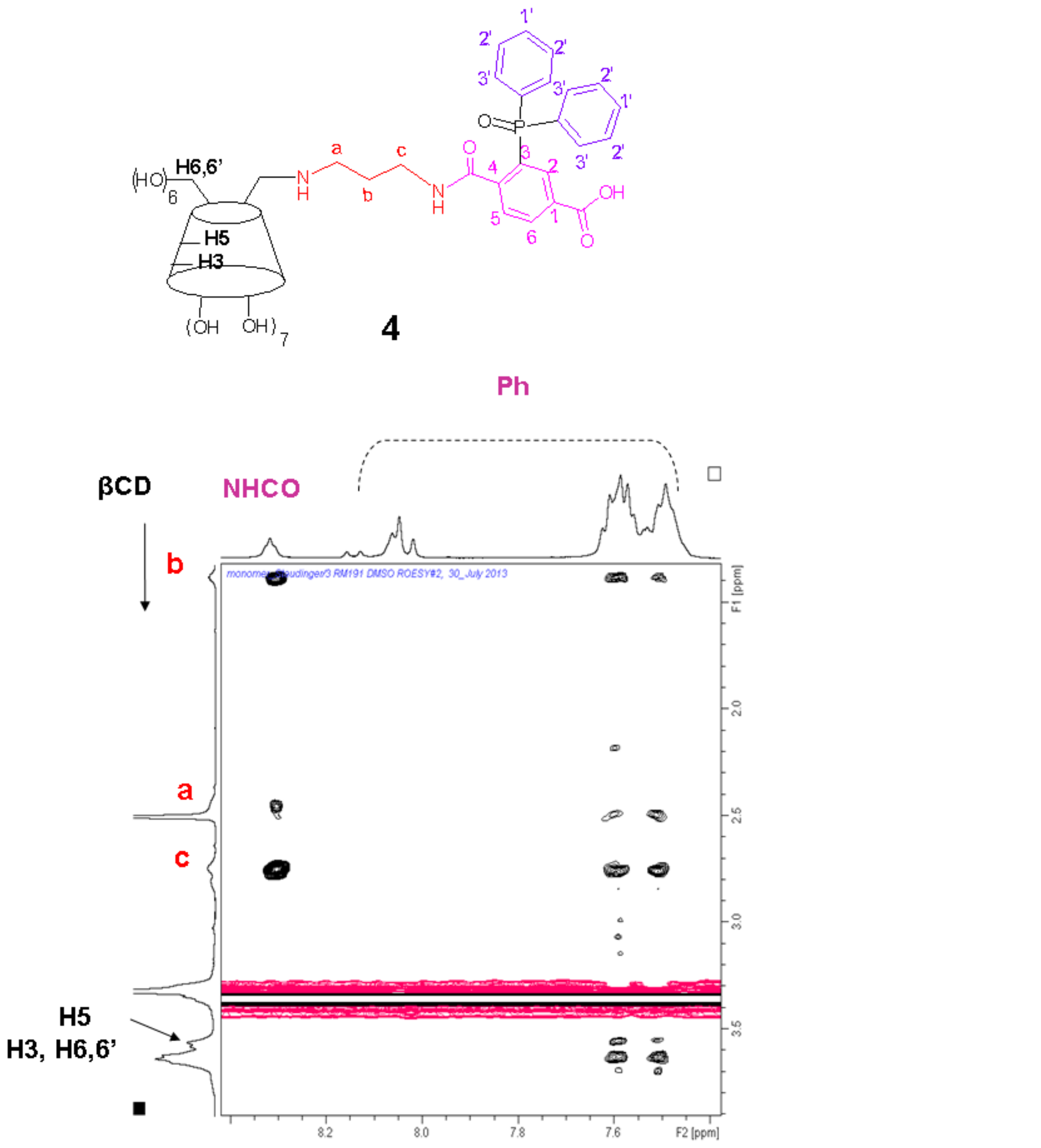

Figure S4: Partial 2D ROESY NMR spectrum of compound **4** in DMSO-d_6 (500 MHz, 298 K): interactions of phenyl protons with primary βCD side protons (H6,6', H5) and with chain protons a, b and c are observed.

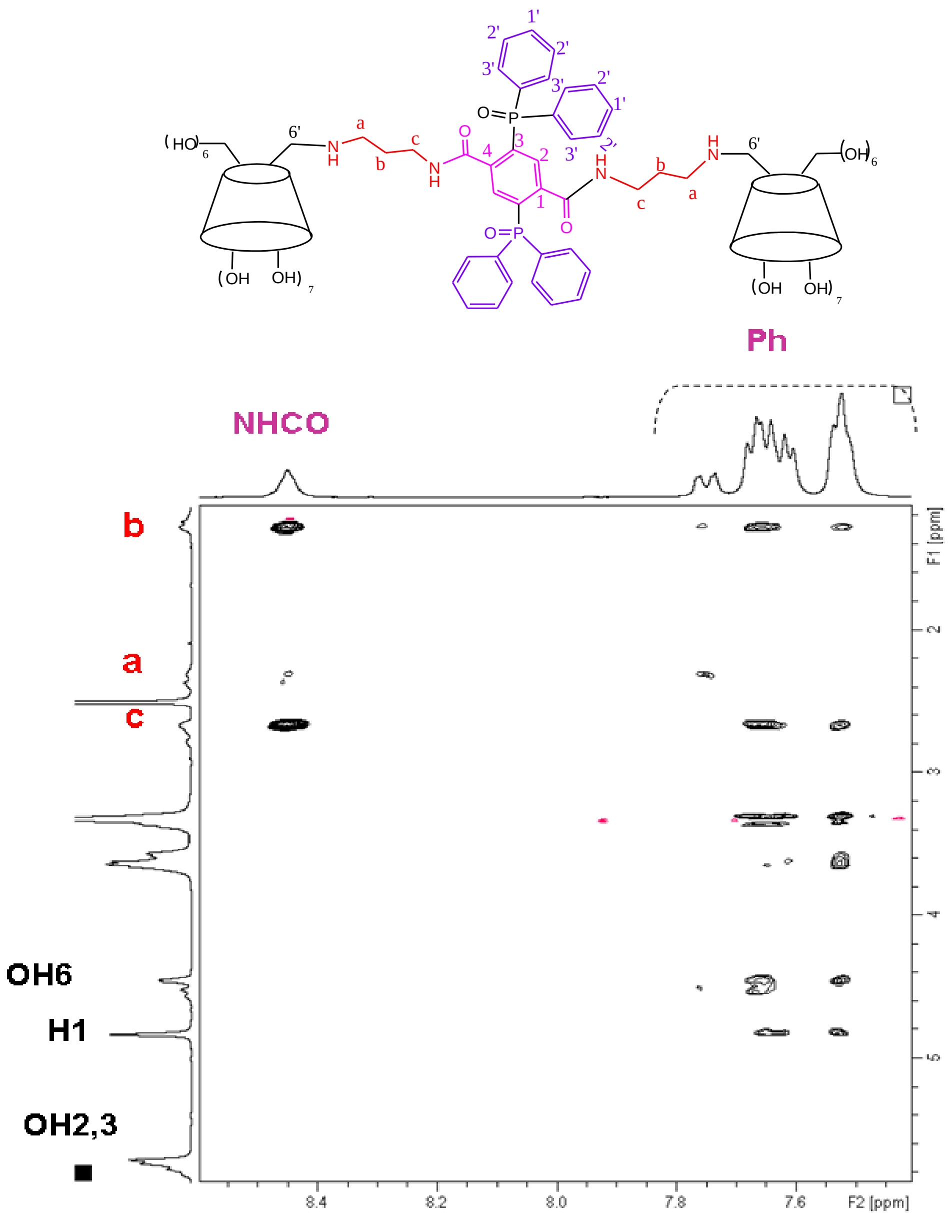

Figure S5: Partial 2D ROESY NMR spectrum of dimer **6** in DMSO-d_6 (500 MHz, 298 K): interactions of phenyl protons with primary βCD side protons (OH6) and with external ones (H1, H2, H4) as well as with chain protons a, b and c are observed but not with wider side groups OH2 and OH3.

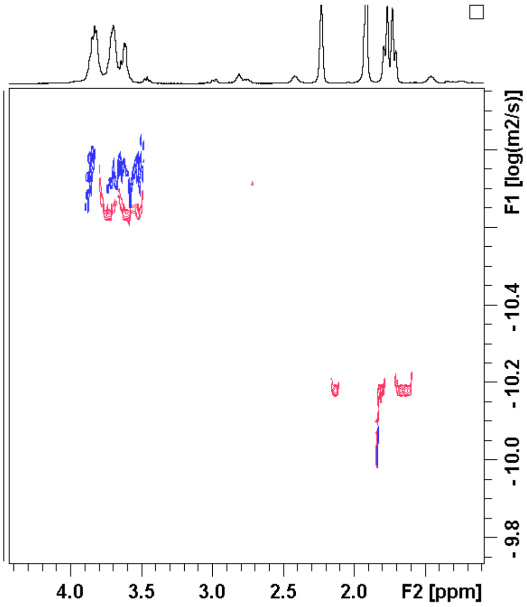

Figure S6: 2D DOSY NMR spectrum of dimer **6** in D_2O alone (1 mM, blue contours) and in the presence of 4 equivalent of 1-adamantylamine·HCl (red contours), 298 K. The water diffusion peak was taken as reference.

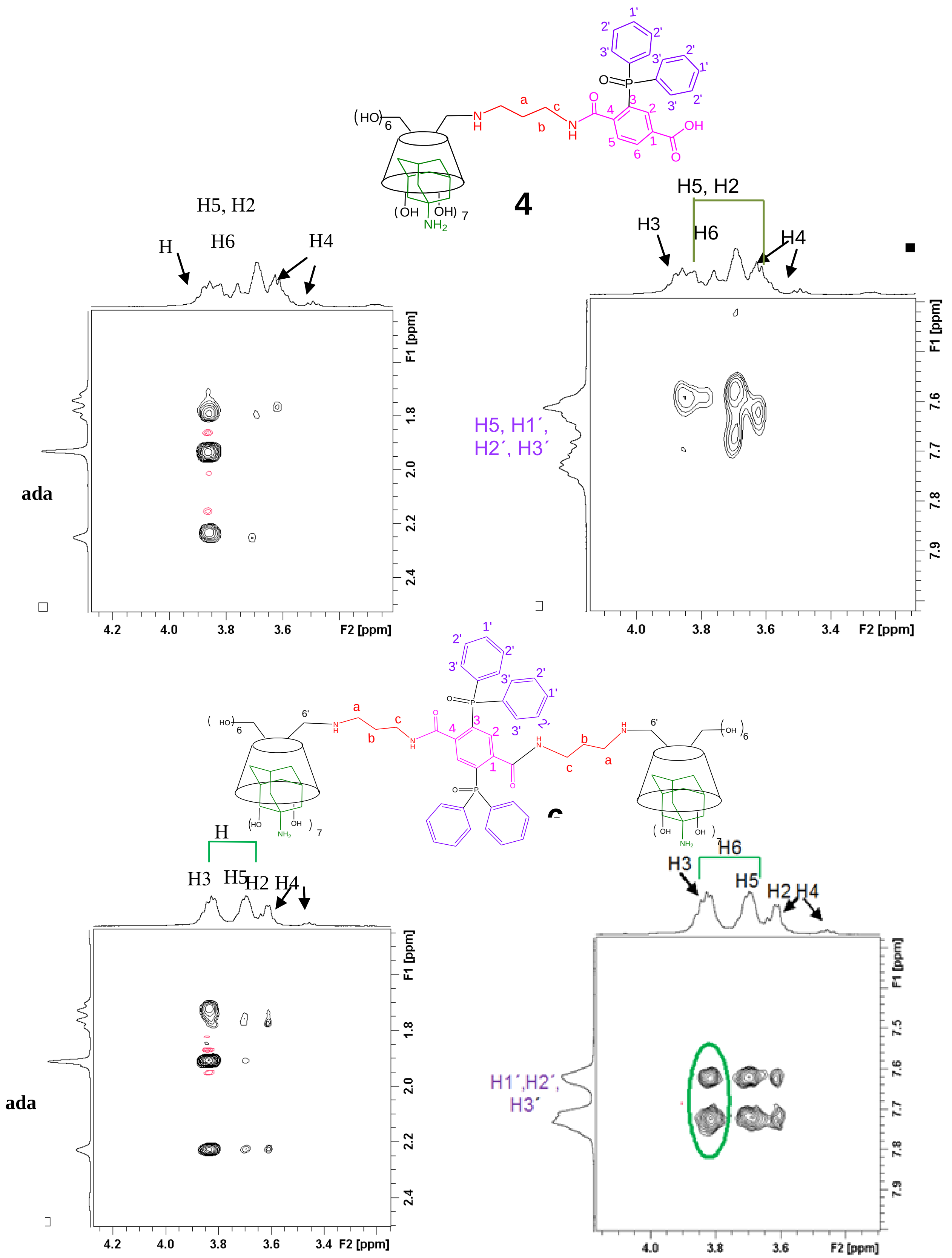

Figure S7: Partial 2D ROESY spectra (D_2O, 500 MHz, 297 K) of the monomer **4** (0.5 mM) with 1 equivalent of 1-adamantylamine·HCl (**ada**), and the dimer **6** (1 mM) with 4 equivalents of **ada**. Left: **ada** vs βCD region; right: phenyl vs βCD region.

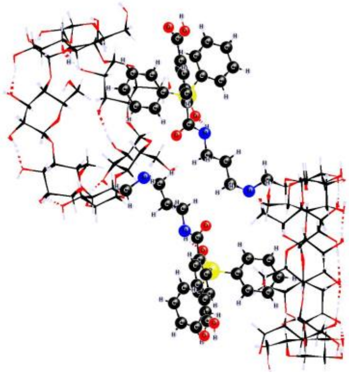

Double Inclusion (Primary side)

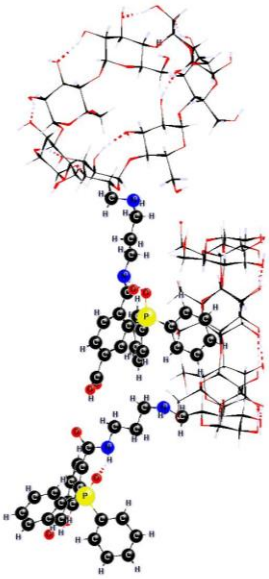

Single Inclusion (Primary side)

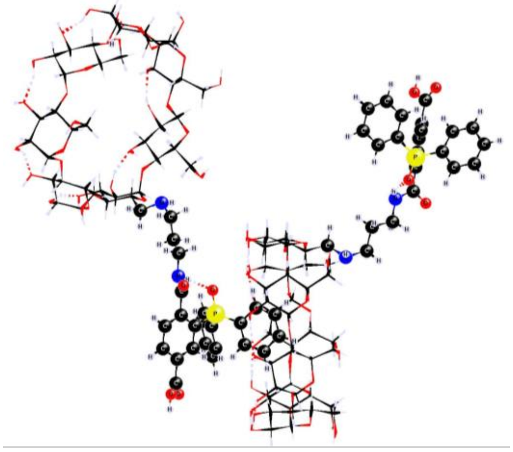

Inclusion (Secondary side)

Figure S8: Geometries of three typical arrangements for a pair of monomers **4** at the PM3(COSMO) level of theory.

Table S1: The minimum distances (Å) between H atoms of the phenyl rings and glucopyranose H atom of βCD for each limiting case of monomer **4** and dimer **6**, as well as for a pair of monomers **4** calculated at the PM3(COSMO) level of theory.[a]

**1. Monomer 4**								
**Type**	**Phenyl**[b]	**CD-H1**	**CD-H2**	**CD-H3**	**CD-H4**	**CD-H5**	**CD-H6**	**CD-H6'**
Inclusion	A	4.77 (7.22)	5.45 (7.65)	**3.00** (5.13)	4.58 (7.03)	**1.70** (4.19)	**3.45** (5.51)	**3.36** (5.62)
	B	4.56 (6.73)	6.27 (7.97)	5.76 (8.00)	4.82 (7.17)	**3.20** (5.61)	**2.56** (4.85)	**1.74** (4.30)
Vicinal	A	4.63 (7.09)	6.34 (8.61)	5.64 (7.52)	4.81 (7.16)	**3.13** (5.03)	**1.72** (4.43)	**1.75** (4.30)
	B	5.65 (7.89)	6.73 (8.93)	6.54 (8.24)	5.43 (7.57)	**3.77** (5.57)	**1.71** (4.21)	**2.89** (4.91)
Open	A	6.71 (8.99)	6.45 (8.71)	9.12 (11.31)	6.54 (8.67)	7.90 (10.50)	6.87 (9.45)	5.17 (7.80)
	B	9.65 (12.13)	10.63 (12.84)	12.37 (14.81)	10.16 (12.38)	10.37 (13.00)	9.26 (11.73)	7.97 (10.40)
**2. Dimer 6**								
**Type**	**Phenyl**[b]	**CD-H1**	**CD-H2**	**CD-H3**	**CD-H4**	**CD-H5**	**CD-H6**	**CD-H6'**
Inclusion/Vicinal	A	4.74 (6.48)	4.75 (6.79)	**1.76** (4.23)	4.19 (6.44)	**1.70** (4.23)	**3.62** (6.55)	**3.35** (5.81)
	B	4.98 (6.20)	6.37 (8.17)	5.01 (6.79)	4.80 (7.61)	**2.58** (4.40)	**1.75** (4.26)	**1.78** (4.71)
	A'	4.91 (7.12)	6.59 (8.46)	5.92 (8.21)	4.97 (7.41)	**3.42** (5.78)	**1.70** (3.99)	**1.76** (4.35)
	B'	4.90 (6.68)	6.33 (7.93)	5.96 (7.92)	4.86 (6.98)	**3.32** (5.29)	**2.52** (3.67)	**1.76** (4.28)
Inclusion	A	4.96 (7.11)	5.37 (7.30)	**2.58** (4.93)	4.51 (6.59)	**1.70** (4.25)	**3.27** (6.23)	**3.26** (5.45)
	B	**3.87** (6.18)	5.47 (7.27)	5.70 (7.65)	4.66 (7.12)	**3.12** (5.01)	**1.72** (4.15)	**1.79** (4.73)
	A'	4.59 (7.05)	5.31 (7.69)	**2.76** (5.08)	4.54 (6.99)	**1.71** (4.63)	**2.75** (5.43)	**3.09** (5.42)
	B'	5.65 (6.46)	6.81 (8.16)	6.19 (7.10)	5.43 (7.15)	**3.55** (5.31)	**1.78** (4.35)	**2.64** (4.39)
Vicinal	A	5.15 (7.04)	6.65 (8.53)	6.65 (8.74)	5.19 (7.35)	**3.91** (6.12)	**1.70** (4.24)	**2.40** (4.65)
	B	7.58 (9.89)	9.52 (11.60)	9.96 (12.49)	7.84 (10.12)	7.43 (10.11)	5.03 (7.69)	5.49 (8.19)
	A'	5.18 (7.06)	6.69 (8.49)	6.57 (8.59)	5.33 (7.59)	**3.89** (6.02)	**1.71** (4.09)	**2.35** (4.72)
	B'	8.12 (10.13)	9.47 (11.76)	9.67 (11.84)	8.03 (10.26)	6.89 (9.13)	5.08 (7.84)	5.80 (8.31)
Open	A	6.87 (9.66)	7.54 (10.02)	9.78 (12.45)	6.78 (9.58)	9.38 (12.20)	8.35 (11.18)	8.12 (11.01)
	B	6.60 (9.48)	7.11 (9.80)	10.01 (12.76)	7.79 (10.62)	9.45 (12.27)	8.47 (11.20)	8.41 (11.27)
	A'	6.20 (8.76)	7.41 (10.17)	9.40 (12.12)	6.40 (9.20)	9.18 (12.00)	8.26 (11.08)	8.03 (10.77)
	B'	8.13 (10.98)	7.13 (10.00)	10.11 (12.94)	7.80 (10.60)	10.18 (13.03)	9.39 (11.51)	9.93 (12.65)
**3. Monomer 4 pair**								
**Type**	**Phenyl**[b]	**CD-H1**	**CD-H2**	**CD-H3**	**CD-H4**	**CD-H5**	**CD-H6**	**CD-H6'**
Single Inclusion (Primary)	A	4.53 (6.86)	5.40 (7.75)	**2.82** (4.95)	4.50 (6.43)	**1.72** (4.26)	**3.49** (6.10)	**3.06** (5.93)
	B	5.79 (6.61)	7.26 (8.11)	6.80 (7.55)	6.06 (6.84)	4.06 (5.08)	**2.41** (4.04)	**3.16** (4.16)

	A':	7.78 (10.20)	7.97 (10.15)	10.01 (12.43)	7.39 (9.67)	8.41 (11.11)	6.98 (9.61)	5.51 (8.17)
	B'	10.25 (12.86)	11.36 (13.77)	12.69 (15.30)	10.67 (13.05)	10.40 (13.11)	9.22 (11.74)	8.22 (10.73)
Double Inclusion (Primary)	A	4.65 (6.53)	5.63 (7.88)	**3.64** (5.82)	4.80 (7.03)	**1.74** (4.32)	**1.75** (4.48)	**1.75** (4.32)
	B	7.09 (9.56)	7.50 (9.75)	8.26 (10.50)	7.43 (9.89)	5.50 (7.93)	4.12 (6.43)	4.59 (6.45)
	A'	4.73 (7.18)	5.59 (7.75)	**3.39** (5.42)	4.61 (7.03)	**1.71** (4.26)	**2.91** (5.18)	**2.50** (4.80)
	B'	6.34 (8.83)	6.72 (8.90)	7.31 (9.72)	6.83 (9.22)	4.52 (7.01)	**3.39** (5.44)	**3.80** (6.67)
Inclusion (Secondary)	A	4.72 (6.61)	4.80 (7.29)	**1.72** (4.31)	4.21 (6.53)	**1.75** (4.10)	**3.95** (6.23)	**3.64** (5.73)
	B	5.67 (7.10)	4.58 (6.67)	**3.28** (5.08)	5.76 (7.83)	5.96 (7.31)	7.59 (10.27)	5.94 (8.62)
	A'	7.01 (9.27)	7.34 (9.48)	9.67 (11.94)	7.16 (9.33)	8.20 (10.82)	7.22 (9.82)	5.55 (8.16)
	B'	10.18 (12.63)	11.34 (13.57)	12.72 (15.19)	10.57 (12.81)	10.54 (13.17)	9.28 (12.09)	8.21 (10.63)

[a]Values in parentheses denote the average distance between the particular glucopyranose H atom with all five H atoms of the nearest phenyl ring. **[b]**A and/or A'= the phenyl inside or on top βCD moiety; B and/or B' = the phenyl outside or farthest from βCD moiety. Average values for all five hydrogen atoms of corresponding phenyl group in parentheses. Close distances (< 4 Å) in **bold**.

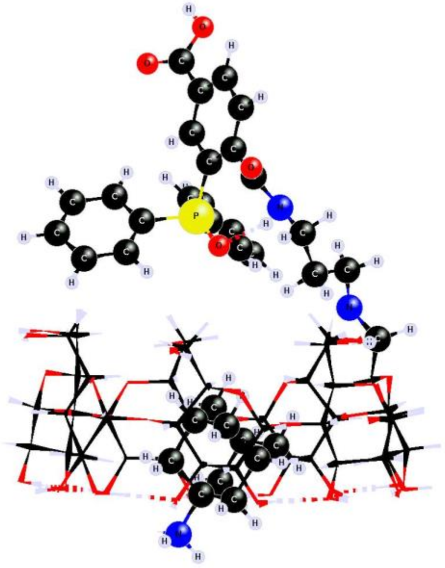

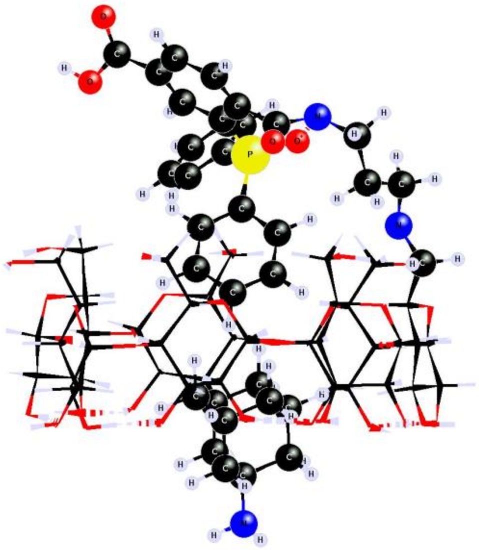

Figure S9: Geometries of the complexes between the vicinal configuration (top) and the inclusion configuration (bottom) of **4** with 1-adamantylammonium at the PM3(COSMO) level of theory.

References

1. Zhou, Q.; Swager, T. *J. Am. Chem. Soc.* **1995,** *117*, 12593-12602.
2. Benin, V.; Durganala, S.; Morgan, A. B. *J. Mater. Chem.* **2012,** *22*, 1180-1190.
3. Brady, B.; Lynam, N.; O'Sullivan, T.; Ahern, C.; Darcy, R. *Org. Synth.* **2000,** *77*, 220-224.
4. Saxon, E.; Bertozzi, C. R. *Science* **2000,** *287*, 2007-2010.
5. Stewart, J. J. P. *MOPAC2012, Version 13.071L*, Stewart Computational Chemistry, Colorado Springs, CO, USA, web: http://OpenMOPAC.net.
6. Stewart, J. J. P. *J. Comput. Chem.* **1989,** *10*, 209 - 220.
7. Klamt, A.; Schüümann, G. *J. Chem. Soc. Perkin Trans. 2* **1993**, 799 - 805.
8. *CRC Handbook of Chemistry and Physics,* CRC Press, Inc.: Boca Raton, Florida, 2001.

9. *Gaussian 03* Revision D.01, Gaussian, Inc, Wallingford CT, 2004
10. a) Becke, A. D. *J. Chem. Phys.* **1993**, *98*, 5648-5652; b) Perdew, J. P. *Phys. Rev. B* **1986**, *33*, 8822-8824
11. Petersson, G. A.; A. Bennett, A.; Tensfeldt, T. G. ; al-Laham, M. A.; Shirley, W. A.; Mantziaris, J. *J. Chem. Phys.* **1988**, *89*, 2193-2218.
